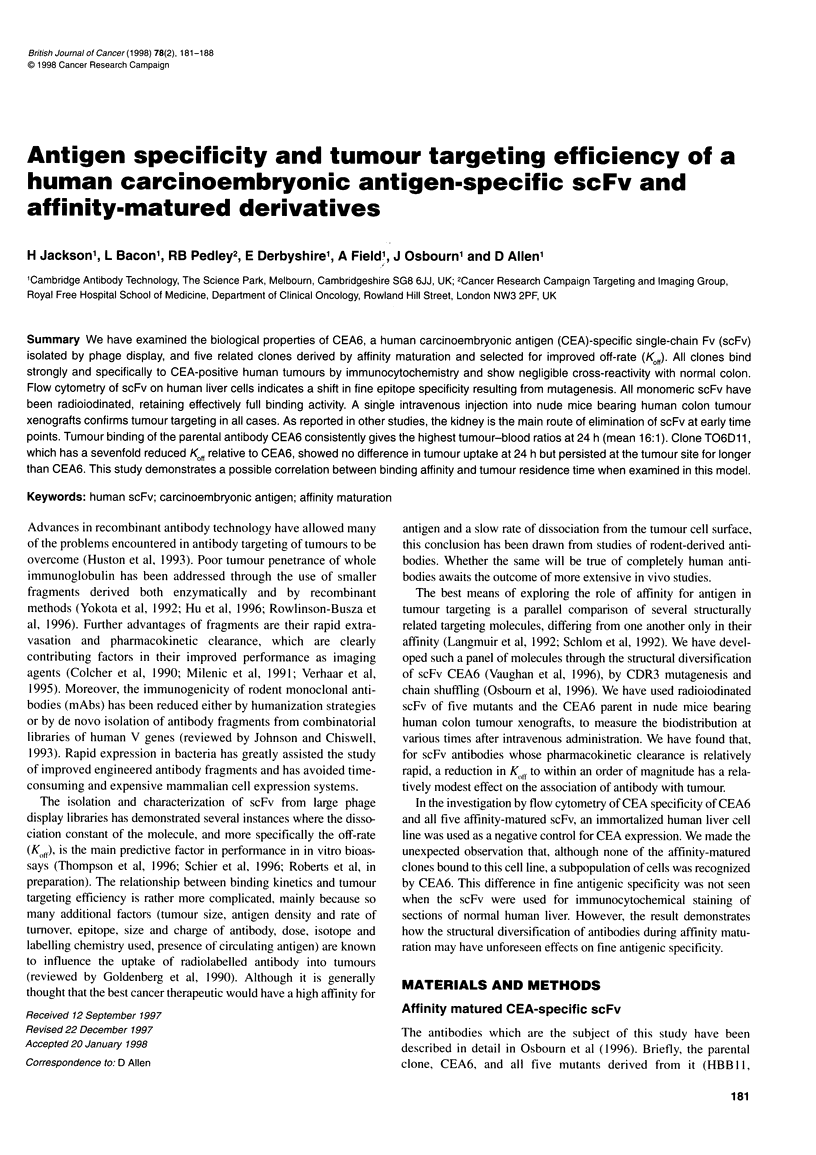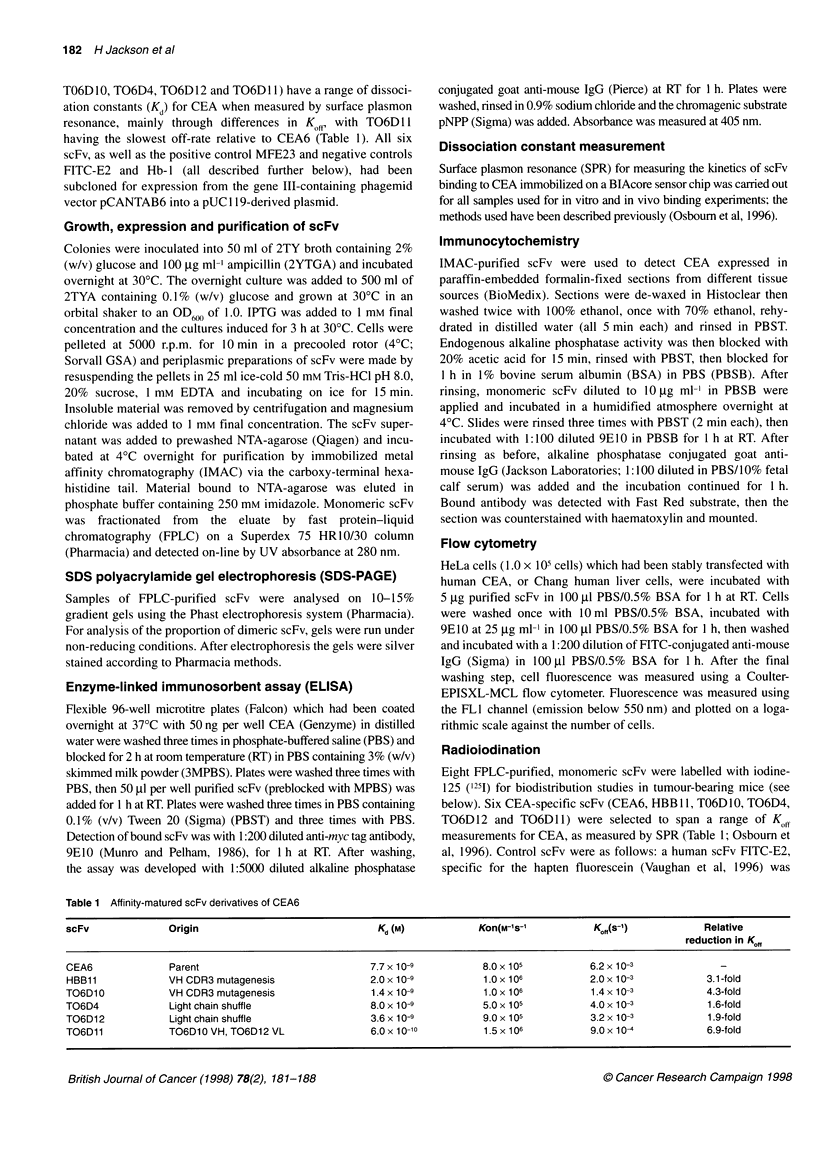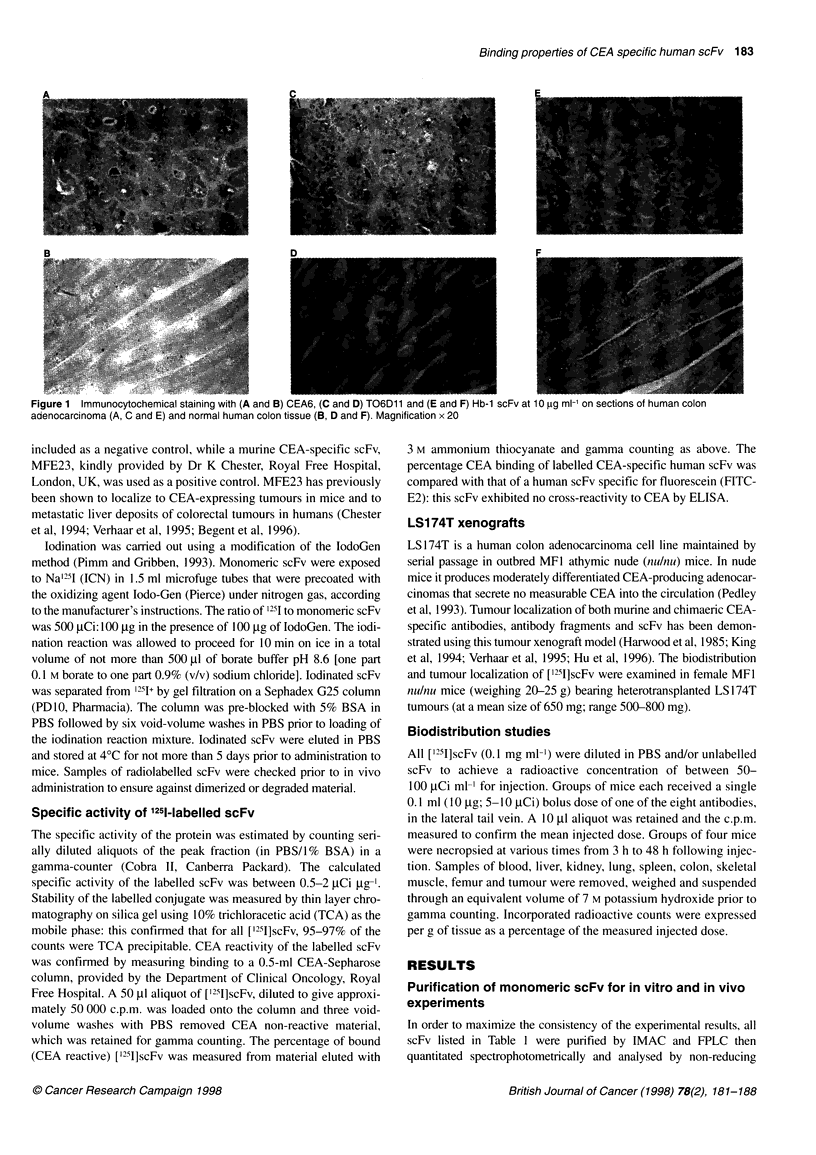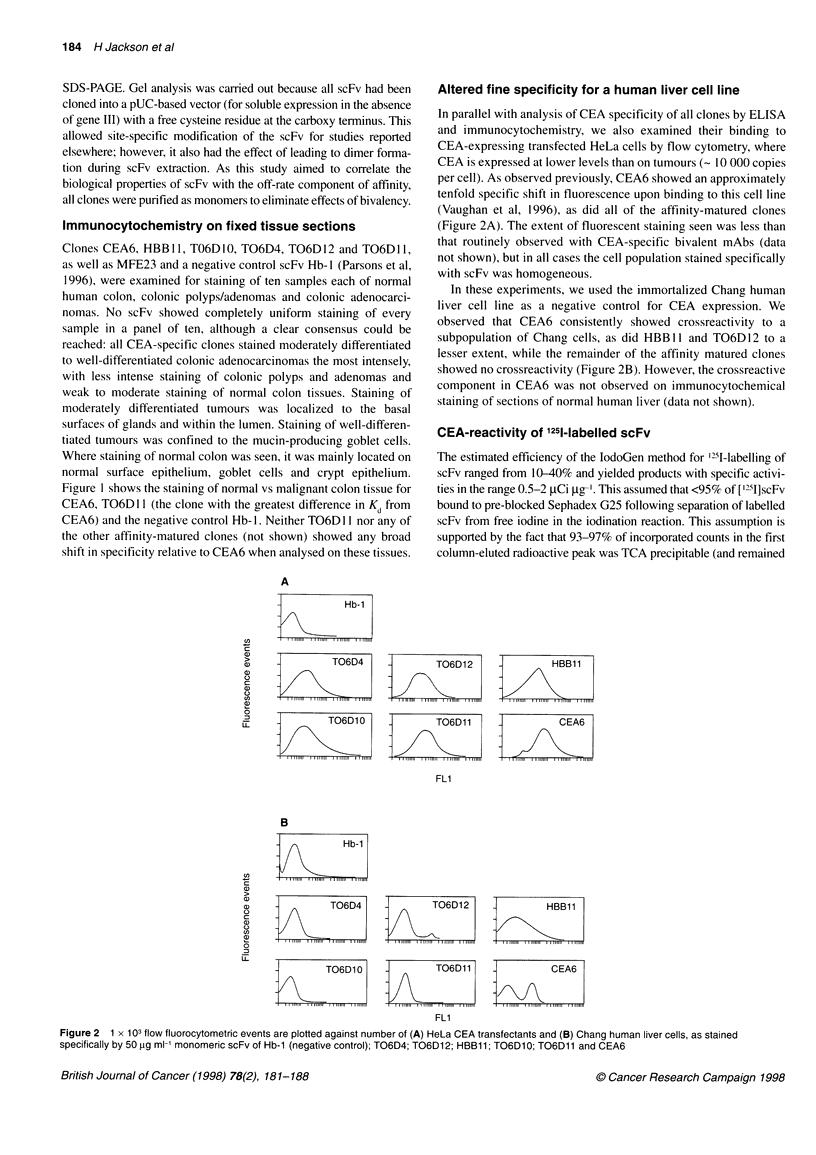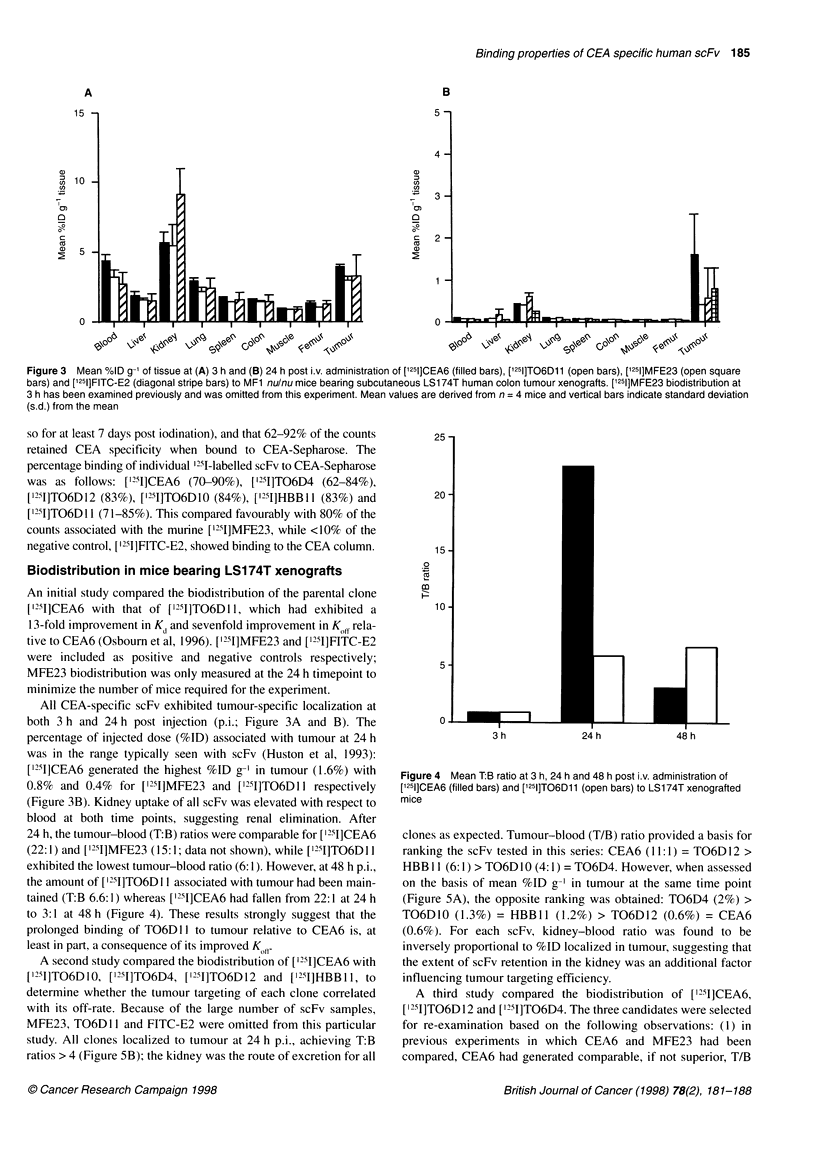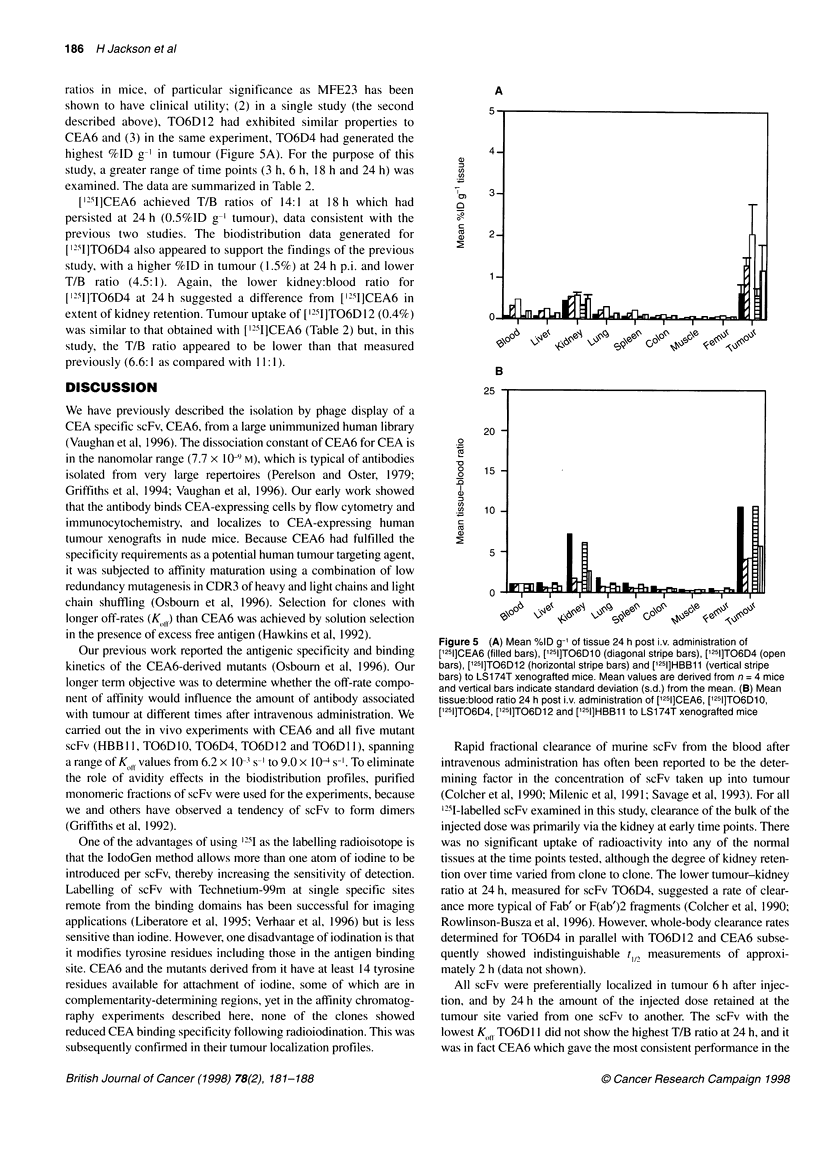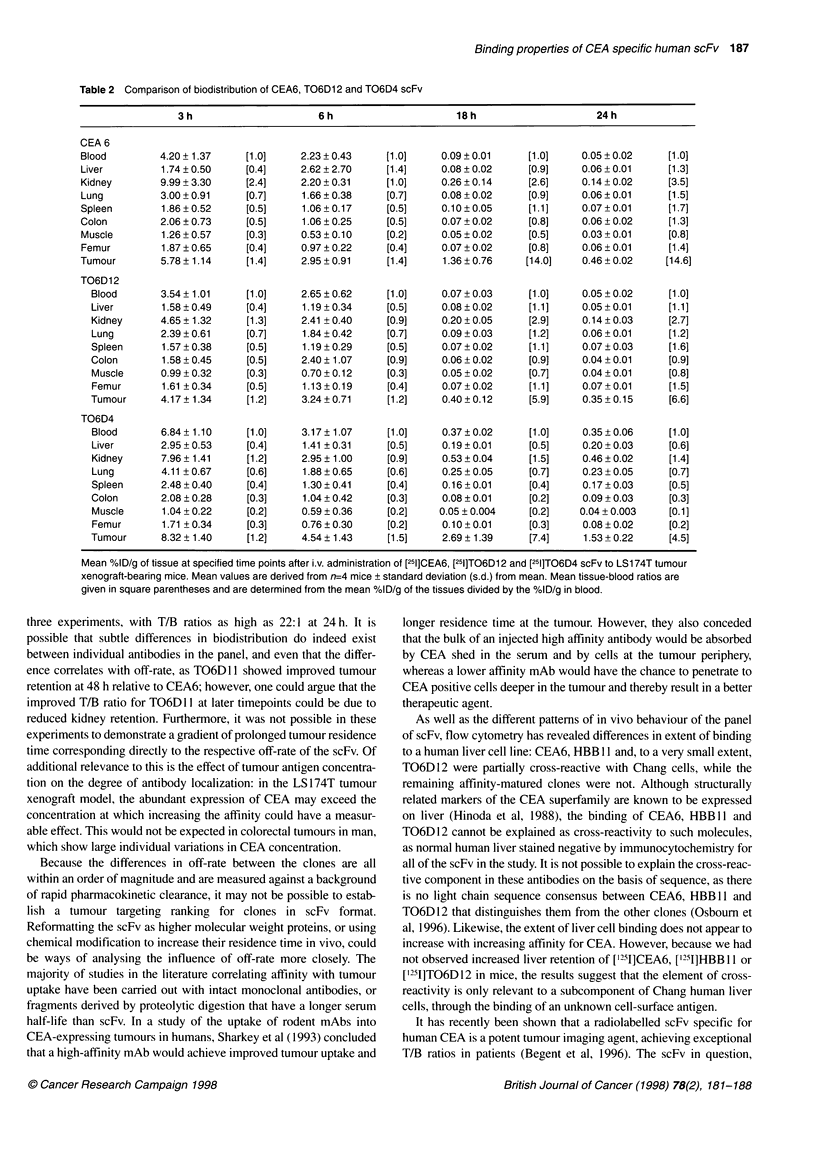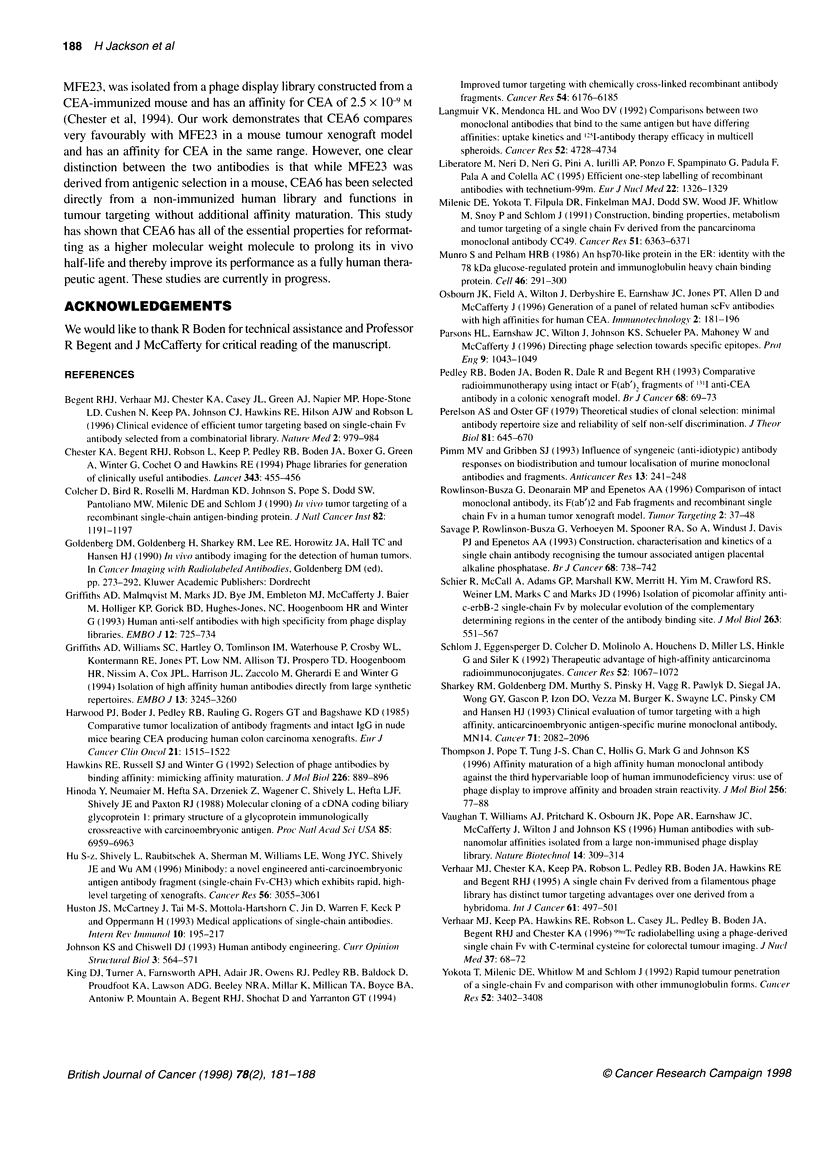# Antigen specificity and tumour targeting efficiency of a human carcinoembryonic antigen-specific scFv and affinity-matured derivatives.

**DOI:** 10.1038/bjc.1998.462

**Published:** 1998-07

**Authors:** H. Jackson, L. Bacon, R. B. Pedley, E. Derbyshire, A. Field, J. Osbourn, D. Allen

**Affiliations:** Cambridge Antibody Technology, The Science Park, Melbourn, Cambridgeshire, UK.

## Abstract

**Images:**


British Journal of Cancer (1998) 78(2), 181–188
© 1998 Cancer Research Campaign

### Antigen specificity and tumour targeting efficiency of a human carcinoembryonic antigen-specific scFv and affinity-matured derivatives

**H Jackson[1], L Bacon[1], RB Pedley[2], E Derbyshire[1], A Field[1], J Osbourn[1] and D Allen[1]**

[1]Cambridge Antibody Technology, The Science Park, Melbourn, Cambridgeshire SG8 6JJ, UK; [2]Cancer Research Campaign Targeting and Imaging Group, Royal Free Hospital School of Medicine, Department of Clinical Oncology, Rowland Hill Street, London NW3 2PF, UK

**Summary** We have examined the biological properties of CEA6, a human carcinoembryonic antigen (CEA)-specific single-chain Fv (scFv) isolated by phage display, and five related clones derived by affinity maturation and selected for improved off-rate ($K_{off}$). All clones bind strongly and specifically to CEA-positive human tumours by immunocytochemistry and show negligible cross-reactivity with normal colon. Flow cytometry of scFv on human liver cells indicates a shift in fine epitope specificity resulting from mutagenesis. All monomeric scFv have been radioiodinated, retaining effectively full binding activity. A single intravenous injection into nude mice bearing human colon tumour xenografts confirms tumour targeting in all cases. As reported in other studies, the kidney is the main route of elimination of scFv at early time points. Tumour binding of the parental antibody CEA6 consistently gives the highest tumour–blood ratios at 24 h (mean 16:1). Clone TO6D11, which has a sevenfold reduced $K_{off}$ relative to CEA6, showed no difference in tumour uptake at 24 h but persisted at the tumour site for longer than CEA6. This study demonstrates a possible correlation between binding affinity and tumour residence time when examined in this model.

**Keywords:** human scFv; carcinoembryonic antigen; affinity maturation

Advances in recombinant antibody technology have allowed many of the problems encountered in antibody targeting of tumours to be overcome (Huston et al, 1993). Poor tumour penetrance of whole immunoglobulin has been addressed through the use of smaller fragments derived both enzymatically and by recombinant methods (Yokota et al, 1992; Hu et al, 1996; Rowlinson-Busza et al, 1996). Further advantages of fragments are their rapid extravasation and pharmacokinetic clearance, which are clearly contributing factors in their improved performance as imaging agents (Colcher et al, 1990; Milenic et al, 1991; Verhaar et al, 1995). Moreover, the immunogenicity of rodent monoclonal antibodies (mAbs) has been reduced either by humanization strategies or by de novo isolation of antibody fragments from combinatorial libraries of human V genes (reviewed by Johnson and Chiswell, 1993). Rapid expression in bacteria has greatly assisted the study of improved engineered antibody fragments and has avoided time-consuming and expensive mammalian cell expression systems.

The isolation and characterization of scFv from large phage display libraries has demonstrated several instances where the dissociation constant of the molecule, and more specifically the off-rate ($K_{off}$), is the main predictive factor in performance in in vitro bioassays (Thompson et al, 1996; Schier et al, 1996; Roberts et al, in preparation). The relationship between binding kinetics and tumour targeting efficiency is rather more complicated, mainly because so many additional factors (tumour size, antigen density and rate of turnover, epitope, size and charge of antibody, dose, isotope and labelling chemistry used, presence of circulating antigen) are known to influence the uptake of radiolabelled antibody into tumours (reviewed by Goldenberg et al, 1990). Although it is generally thought that the best cancer therapeutic would have a high affinity for antigen and a slow rate of dissociation from the tumour cell surface, this conclusion has been drawn from studies of rodent-derived antibodies. Whether the same will be true of completely human antibodies awaits the outcome of more extensive in vivo studies.

Received 12 September 1997
Revised 22 December 1997
Accepted 20 January 1998

Correspondence to: D Allen

The best means of exploring the role of affinity for antigen in tumour targeting is a parallel comparison of several structurally related targeting molecules, differing from one another only in their affinity (Langmuir et al, 1992; Schlom et al, 1992). We have developed such a panel of molecules through the structural diversification of scFv CEA6 (Vaughan et al, 1996), by CDR3 mutagenesis and chain shuffling (Osbourn et al, 1996). We have used radioiodinated scFv of five mutants and the CEA6 parent in nude mice bearing human colon tumour xenografts, to measure the biodistribution at various times after intravenous administration. We have found that, for scFv antibodies whose pharmacokinetic clearance is relatively rapid, a reduction in $K_{off}$ to within an order of magnitude has a relatively modest effect on the association of antibody with tumour.

In the investigation by flow cytometry of CEA specificity of CEA6 and all five affinity-matured scFv, an immortalized human liver cell line was used as a negative control for CEA expression. We made the unexpected observation that, although none of the affinity-matured clones bound to this cell line, a subpopulation of cells was recognized by CEA6. This difference in fine antigenic specificity was not seen when the scFv were used for immunocytochemical staining of sections of normal human liver. However, the result demonstrates how the structural diversification of antibodies during affinity maturation may have unforeseen effects on fine antigenic specificity.

### MATERIALS AND METHODS

### Affinity matured CEA-specific scFv

The antibodies which are the subject of this study have been described in detail in Osbourn et al (1996). Briefly, the parental clone, CEA6, and all five mutants derived from it (HBB11,

T06D10, T06D4, T06D12 and T06D11) have a range of dissociation constants ($K_d$) for CEA when measured by surface plasmon resonance, mainly through differences in $K_{off}$, with T06D11 having the slowest off-rate relative to CEA6 (Table 1). All six scFv, as well as the positive control MFE23 and negative controls FITC-E2 and Hb-1 (all described further below), had been subcloned for expression from the gene III-containing phagemid vector pCANTAB6 into a pUC119-derived plasmid.

### Growth, expression and purification of scFv

Colonies were inoculated into 50 ml of 2TY broth containing 2% (w/v) glucose and 100 µg ml$^{-1}$ ampicillin (2YTGA) and incubated overnight at 30°C. The overnight culture was added to 500 ml of 2TYA containing 0.1% (w/v) glucose and grown at 30°C in an orbital shaker to an $OD_{600}$ of 1.0. IPTG was added to 1 mM final concentration and the cultures induced for 3 h at 30°C. Cells were pelleted at 5000 r.p.m. for 10 min in a precooled rotor (4°C; Sorvall GSA) and periplasmic preparations of scFv were made by resuspending the pellets in 25 ml ice-cold 50 mM Tris-HCl pH 8.0, 20% sucrose, 1 mM EDTA and incubating on ice for 15 min. Insoluble material was removed by centrifugation and magnesium chloride was added to 1 mM final concentration. The scFv supernatant was added to prewashed NTA-agarose (Qiagen) and incubated at 4°C overnight for purification by immobilized metal affinity chromatography (IMAC) via the carboxy-terminal hexahistidine tail. Material bound to NTA-agarose was eluted in phosphate buffer containing 250 mM imidazole. Monomeric scFv was fractionated from the eluate by fast protein–liquid chromatography (FPLC) on a Superdex 75 HR10/30 column (Pharmacia) and detected on-line by UV absorbance at 280 nm.

### SDS polyacrylamide gel electrophoresis (SDS-PAGE)

Samples of FPLC-purified scFv were analysed on 10–15% gradient gels using the Phast electrophoresis system (Pharmacia). For analysis of the proportion of dimeric scFv, gels were run under non-reducing conditions. After electrophoresis the gels were silver stained according to Pharmacia methods.

### Enzyme-linked immunosorbent assay (ELISA)

Flexible 96-well microtitre plates (Falcon) which had been coated overnight at 37°C with 50 ng per well CEA (Genzyme) in distilled water were washed three times in phosphate-buffered saline (PBS) and blocked for 2 h at room temperature (RT) in PBS containing 3% (w/v) skimmed milk powder (3MPBS). Plates were washed three times with PBS, then 50 µl per well purified scFv (preblocked with MPBS) was added for 1 h at RT. Plates were washed three times in PBS containing 0.1% (v/v) Tween 20 (Sigma) (PBST) and three times with PBS. Detection of bound scFv was with 1:200 diluted anti-*myc* tag antibody, 9E10 (Munro and Pelham, 1986), for 1 h at RT. After washing, the assay was developed with 1:5000 diluted alkaline phosphatase conjugated goat anti-mouse IgG (Pierce) at RT for 1 h. Plates were washed, rinsed in 0.9% sodium chloride and the chromagenic substrate pNPP (Sigma) was added. Absorbance was measured at 405 nm.

### Dissociation constant measurement

Surface plasmon resonance (SPR) for measuring the kinetics of scFv binding to CEA immobilized on a BIAcore sensor chip was carried out for all samples used for in vitro and in vivo binding experiments; the methods used have been described previously (Osbourn et al, 1996).

### Immunocytochemistry

IMAC-purified scFv were used to detect CEA expressed in paraffin-embedded formalin-fixed sections from different tissue sources (BioMedix). Sections were de-waxed in Histoclear then washed twice with 100% ethanol, once with 70% ethanol, rehydrated in distilled water (all 5 min each) and rinsed in PBST. Endogenous alkaline phosphatase activity was then blocked with 20% acetic acid for 15 min, rinsed with PBST, then blocked for 1 h in 1% bovine serum albumin (BSA) in PBS (PBSB). After rinsing, monomeric scFv diluted to 10 µg ml$^{-1}$ in PBSB were applied and incubated in a humidified atmosphere overnight at 4°C. Slides were rinsed three times with PBST (2 min each), then incubated with 1:100 diluted 9E10 in PBSB for 1 h at RT. After rinsing as before, alkaline phosphatase conjugated goat anti-mouse IgG (Jackson Laboratories; 1:100 diluted in PBS/10% fetal calf serum) was added and the incubation continued for 1 h. Bound antibody was detected with Fast Red substrate, then the section was counterstained with haematoxylin and mounted.

### Flow cytometry

HeLa cells ($1.0 \times 10^5$ cells) which had been stably transfected with human CEA, or Chang human liver cells, were incubated with 5 µg purified scFv in 100 µl PBS/0.5% BSA for 1 h at RT. Cells were washed once with 10 ml PBS/0.5% BSA, incubated with 9E10 at 25 µg ml$^{-1}$ in 100 µl PBS/0.5% BSA for 1 h, then washed and incubated with a 1:200 dilution of FITC-conjugated anti-mouse IgG (Sigma) in 100 µl PBS/0.5% BSA for 1 h. After the final washing step, cell fluorescence was measured using a Coulter-EPISXL-MCL flow cytometer. Fluorescence was measured using the FL1 channel (emission below 550 nm) and plotted on a logarithmic scale against the number of cells.

### Radioiodination

Eight FPLC-purified, monomeric scFv were labelled with iodine-125 ($^{125}I$) for biodistribution studies in tumour-bearing mice (see below). Six CEA-specific scFv (CEA6, HBB11, T06D10, T06D4, T06D12 and T06D11) were selected to span a range of $K_{off}$ measurements for CEA, as measured by SPR (Table 1; Osbourn et al, 1996). Control scFv were as follows: a human scFv FITC-E2, specific for the hapten fluorescein (Vaughan et al, 1996) was

**Table 1** Affinity-matured scFv derivatives of CEA6

| scFv | Origin | $K_d$ (M) | Kon(M$^{-1}$s$^{-1}$ | $K_{off}$(s$^{-1}$) | Relative reduction in $K_{off}$ |
|---|---|---|---|---|---|
| CEA6 | Parent | $7.7 \times 10^{-9}$ | $8.0 \times 10^5$ | $6.2 \times 10^{-3}$ | – |
| HBB11 | VH CDR3 mutagenesis | $2.0 \times 10^{-9}$ | $1.0 \times 10^6$ | $2.0 \times 10^{-3}$ | 3.1-fold |
| TO6D10 | VH CDR3 mutagenesis | $1.4 \times 10^{-9}$ | $1.0 \times 10^6$ | $1.4 \times 10^{-3}$ | 4.3-fold |
| TO6D4 | Light chain shuffle | $8.0 \times 10^{-9}$ | $5.0 \times 10^5$ | $4.0 \times 10^{-3}$ | 1.6-fold |
| TO6D12 | Light chain shuffle | $3.6 \times 10^{-9}$ | $9.0 \times 10^5$ | $3.2 \times 10^{-3}$ | 1.9-fold |
| TO6D11 | TO6D10 VH, TO6D12 VL | $6.0 \times 10^{-10}$ | $1.5 \times 10^6$ | $9.0 \times 10^{-4}$ | 6.9-fold |

 © Cancer Research Campaign 1998

**Figure 1** Immunocytochemical staining with (**A** and **B**) CEA6, (**C** and **D**) TO6D11 and (**E** and **F**) Hb-1 scFv at 10 μg ml$^{-1}$ on sections of human colon adenocarcinoma (A, C and E) and normal human colon tissue (**B**, **D** and **F**). Magnification × 20

included as a negative control, while a murine CEA-specific scFv, MFE23, kindly provided by Dr K Chester, Royal Free Hospital, London, UK, was used as a positive control. MFE23 has previously been shown to localize to CEA-expressing tumours in mice and to metastatic liver deposits of colorectal tumours in humans (Chester et al, 1994; Verhaar et al, 1995; Begent et al, 1996).

Iodination was carried out using a modification of the IodoGen method (Pimm and Gribben, 1993). Monomeric scFv were exposed to Na$^{125}$I (ICN) in 1.5 ml microfuge tubes that were precoated with the oxidizing agent Iodo-Gen (Pierce) under nitrogen gas, according to the manufacturer's instructions. The ratio of $^{125}$I to monomeric scFv was 500 μCi:100 μg in the presence of 100 μg of IodoGen. The iodination reaction was allowed to proceed for 10 min on ice in a total volume of not more than 500 μl of borate buffer pH 8.6 [one part 0.1 M borate to one part 0.9% (v/v) sodium chloride]. Iodinated scFv was separated from $^{125}$I$^{+}$ by gel filtration on a Sephadex G25 column (PD10, Pharmacia). The column was pre-blocked with 5% BSA in PBS followed by six void-volume washes in PBS prior to loading of the iodination reaction mixture. Iodinated scFv were eluted in PBS and stored at 4°C for not more than 5 days prior to administration to mice. Samples of radiolabelled scFv were checked prior to in vivo administration to ensure against dimerized or degraded material.

### Specific activity of $^{125}$I-labelled scFv

The specific activity of the protein was estimated by counting serially diluted aliquots of the peak fraction (in PBS/1% BSA) in a gamma-counter (Cobra II, Canberra Packard). The calculated specific activity of the labelled scFv was between 0.5–2 μCi μg$^{-1}$. Stability of the labelled conjugate was measured by thin layer chromatography on silica gel using 10% trichloracetic acid (TCA) as the mobile phase: this confirmed that for all [$^{125}$I]scFv, 95–97% of the counts were TCA precipitable. CEA reactivity of the labelled scFv was confirmed by measuring binding to a 0.5-ml CEA-Sepharose column, provided by the Department of Clinical Oncology, Royal Free Hospital. A 50 μl aliquot of [$^{125}$I]scFv, diluted to give approximately 50 000 c.p.m. was loaded onto the column and three void-volume washes with PBS removed CEA non-reactive material, which was retained for gamma counting. The percentage of bound (CEA reactive) [$^{125}$I]scFv was measured from material eluted with 3 M ammonium thiocyanate and gamma counting as above. The percentage CEA binding of labelled CEA-specific human scFv was compared with that of a human scFv specific for fluorescein (FITC-E2): this scFv exhibited no cross-reactivity to CEA by ELISA.

### LS174T xenografts

LS174T is a human colon adenocarcinoma cell line maintained by serial passage in outbred MF1 athymic nude (*nu/nu*) mice. In nude mice it produces moderately differentiated CEA-producing adenocarcinomas that secrete no measurable CEA into the circulation (Pedley et al, 1993). Tumour localization of both murine and chimaeric CEA-specific antibodies, antibody fragments and scFv has been demonstrated using this tumour xenograft model (Harwood et al, 1985; King et al, 1994; Verhaar et al, 1995; Hu et al, 1996). The biodistribution and tumour localization of [$^{125}$I]scFv were examined in female MF1 *nu/nu* mice (weighing 20–25 g) bearing heterotransplanted LS174T tumours (at a mean size of 650 mg; range 500–800 mg).

### Biodistribution studies

All [$^{125}$I]scFv (0.1 mg ml$^{-1}$) were diluted in PBS and/or unlabelled scFv to achieve a radioactive concentration of between 50–100 μCi ml$^{-1}$ for injection. Groups of mice each received a single 0.1 ml (10 μg; 5–10 μCi) bolus dose of one of the eight antibodies, in the lateral tail vein. A 10 μl aliquot was retained and the c.p.m. measured to confirm the mean injected dose. Groups of four mice were necropsied at various times from 3 h to 48 h following injection. Samples of blood, liver, kidney, lung, spleen, colon, skeletal muscle, femur and tumour were removed, weighed and suspended through an equivalent volume of 7 M potassium hydroxide prior to gamma counting. Incorporated radioactive counts were expressed per g of tissue as a percentage of the measured injected dose.

### RESULTS

### Purification of monomeric scFv for in vitro and in vivo experiments

In order to maximize the consistency of the experimental results, all scFv listed in Table 1 were purified by IMAC and FPLC then quantitated spectrophotometrically and analysed by non-reducing

© Cancer Research Campaign 1998

SDS-PAGE. Gel analysis was carried out because all scFv had been cloned into a pUC-based vector (for soluble expression in the absence of gene III) with a free cysteine residue at the carboxy terminus. This allowed site-specific modification of the scFv for studies reported elsewhere; however, it also had the effect of leading to dimer formation during scFv extraction. As this study aimed to correlate the biological properties of scFv with the off-rate component of affinity, all clones were purified as monomers to eliminate effects of bivalency.

### Immunocytochemistry on fixed tissue sections

Clones CEA6, HBB11, TO6D10, TO6D4, TO6D12 and TO6D11, as well as MFE23 and a negative control scFv Hb-1 (Parsons et al, 1996), were examined for staining of ten samples each of normal human colon, colonic polyps/adenomas and colonic adenocarcinomas. No scFv showed completely uniform staining of every sample in a panel of ten, although a clear consensus could be reached: all CEA-specific clones stained moderately differentiated to well-differentiated colonic adenocarcinomas the most intensely, with less intense staining of colonic polyps and adenomas and weak to moderate staining of normal colon tissues. Staining of moderately differentiated tumours was localized to the basal surfaces of glands and within the lumen. Staining of well-differentiated tumours was confined to the mucin-producing goblet cells. Where staining of normal colon was seen, it was mainly located on normal surface epithelium, goblet cells and crypt epithelium. Figure 1 shows the staining of normal vs malignant colon tissue for CEA6, TO6D11 (the clone with the greatest difference in $K_d$ from CEA6) and the negative control Hb-1. Neither TO6D11 nor any of the other affinity-matured clones (not shown) showed any broad shift in specificity relative to CEA6 when analysed on these tissues.

### Altered fine specificity for a human liver cell line

In parallel with analysis of CEA specificity of all clones by ELISA and immunocytochemistry, we also examined their binding to CEA-expressing transfected HeLa cells by flow cytometry, where CEA is expressed at lower levels than on tumours (~ 10 000 copies per cell). As observed previously, CEA6 showed an approximately tenfold specific shift in fluorescence upon binding to this cell line (Vaughan et al, 1996), as did all of the affinity-matured clones (Figure 2A). The extent of fluorescent staining seen was less than that routinely observed with CEA-specific bivalent mAbs (data not shown), but in all cases the cell population stained specifically with scFv was homogeneous.

In these experiments, we used the immortalized Chang human liver cell line as a negative control for CEA expression. We observed that CEA6 consistently showed crossreactivity to a subpopulation of Chang cells, as did HBB11 and TO6D12 to a lesser extent, while the remainder of the affinity matured clones showed no crossreactivity (Figure 2B). However, the crossreactive component in CEA6 was not observed on immunocytochemical staining of sections of normal human liver (data not shown).

### CEA-reactivity of $^{125}$I-labelled scFv

The estimated efficiency of the IodoGen method for $^{125}$I-labelling of scFv ranged from 10–40% and yielded products with specific activities in the range 0.5–2 μCi μg$^{-1}$. This assumed that <95% of [$^{125}$I]scFv bound to pre-blocked Sephadex G25 following separation of labelled scFv from free iodine in the iodination reaction. This assumption is supported by the fact that 93–97% of incorporated counts in the first column-eluted radioactive peak was TCA precipitable (and remained

**Figure 2** 1 × 10$^3$ flow fluorocytometric events are plotted against number of (**A**) HeLa CEA transfectants and (**B**) Chang human liver cells, as stained specifically by 50 μg ml$^{-1}$ monomeric scFv of Hb-1 (negative control); TO6D4; TO6D12; HBB11; TO6D10; TO6D11 and CEA6

 © Cancer Research Campaign 1998

**Figure 3** Mean %ID g$^{-1}$ of tissue at (**A**) 3 h and (**B**) 24 h post i.v. administration of [$^{125}$I]CEA6 (filled bars), [$^{125}$I]TO6D11 (open bars), [$^{125}$I]MFE23 (open square bars) and [$^{125}$I]FITC-E2 (diagonal stripe bars) to MF1 *nu/nu* mice bearing subcutaneous LS174T human colon tumour xenografts. [$^{125}$I]MFE23 biodistribution at 3 h has been examined previously and was omitted from this experiment. Mean values are derived from $n$ = 4 mice and vertical bars indicate standard deviation (s.d.) from the mean so for at least 7 days post iodination), and that 62–92% of the counts retained CEA specificity when bound to CEA-Sepharose. The percentage binding of individual $^{125}$I-labelled scFv to CEA-Sepharose was as follows: [$^{125}$I]CEA6 (70–90%), [$^{125}$I]TO6D4 (62–84%), [$^{125}$I]TO6D12 (83%), [$^{125}$I]TO6D10 (84%), [$^{125}$I]HBB11 (83%) and [$^{125}$I]TO6D11 (71–85%). This compared favourably with 80% of the counts associated with the murine [$^{125}$I]MFE23, while <10% of the negative control, [$^{125}$I]FITC-E2, showed binding to the CEA column.

### Biodistribution in mice bearing LS174T xenografts

An initial study compared the biodistribution of the parental clone [$^{125}$I]CEA6 with that of [$^{125}$I]TO6D11, which had exhibited a 13-fold improvement in $K_d$ and sevenfold improvement in $K_{off}$ relative to CEA6 (Osbourn et al, 1996). [$^{125}$I]MFE23 and [$^{125}$I]FITC-E2 were included as positive and negative controls respectively; MFE23 biodistribution was only measured at the 24 h timepoint to minimize the number of mice required for the experiment.

All CEA-specific scFv exhibited tumour-specific localization at both 3 h and 24 h post injection (p.i.; Figure 3A and B). The percentage of injected dose (%ID) associated with tumour at 24 h was in the range typically seen with scFv (Huston et al, 1993): [$^{125}$I]CEA6 generated the highest %ID g$^{-1}$ in tumour (1.6%) with 0.8% and 0.4% for [$^{125}$I]MFE23 and [$^{125}$I]TO6D11 respectively (Figure 3B). Kidney uptake of all scFv was elevated with respect to blood at both time points, suggesting renal elimination. After 24 h, the tumour–blood (T:B) ratios were comparable for [$^{125}$I]CEA6 (22:1) and [$^{125}$I]MFE23 (15:1; data not shown), while [$^{125}$I]TO6D11 exhibited the lowest tumour–blood ratio (6:1). However, at 48 h p.i., the amount of [$^{125}$I]TO6D11 associated with tumour had been maintained (T:B 6.6:1) whereas [$^{125}$I]CEA6 had fallen from 22:1 at 24 h to 3:1 at 48 h (Figure 4). These results strongly suggest that the prolonged binding of TO6D11 to tumour relative to CEA6 is, at least in part, a consequence of its improved $K_{off}$.

A second study compared the biodistribution of [$^{125}$I]CEA6 with [$^{125}$I]TO6D10, [$^{125}$I]TO6D4, [$^{125}$I]TO6D12 and [$^{125}$I]HBB11, to determine whether the tumour targeting of each clone correlated with its off-rate. Because of the large number of scFv samples, MFE23, TO6D11 and FITC-E2 were omitted from this particular study. All clones localized to tumour at 24 h p.i., achieving T:B ratios > 4 (Figure 5B); the kidney was the route of excretion for all

**Figure 4** Mean T:B ratio at 3 h, 24 h and 48 h post i.v. administration of [$^{125}$I]CEA6 (filled bars) and [$^{125}$I]TO6D11 (open bars) to LS174T xenografted mice clones as expected. Tumour–blood (T/B) ratio provided a basis for ranking the scFv tested in this series: CEA6 (11:1) = TO6D12 > HBB11 (6:1) > TO6D10 (4:1) = TO6D4. However, when assessed on the basis of mean %ID g$^{-1}$ in tumour at the same time point (Figure 5A), the opposite ranking was obtained: TO6D4 (2%) > TO6D10 (1.3%) = HBB11 (1.2%) > TO6D12 (0.6%) = CEA6 (0.6%). For each scFv, kidney–blood ratio was found to be inversely proportional to %ID localized in tumour, suggesting that the extent of scFv retention in the kidney was an additional factor influencing tumour targeting efficiency.

A third study compared the biodistribution of [$^{125}$I]CEA6, [$^{125}$I]TO6D12 and [$^{125}$I]TO6D4. The three candidates were selected for re-examination based on the following observations: (1) in previous experiments in which CEA6 and MFE23 had been compared, CEA6 had generated comparable, if not superior, T/B

© Cancer Research Campaign 1998 

ratios in mice, of particular significance as MFE23 has been shown to have clinical utility; (2) in a single study (the second described above), TO6D12 had exhibited similar properties to CEA6 and (3) in the same experiment, TO6D4 had generated the highest %ID $g^{-1}$ in tumour (Figure 5A). For the purpose of this study, a greater range of time points (3 h, 6 h, 18 h and 24 h) was examined. The data are summarized in Table 2.

[$^{125}$I]CEA6 achieved T/B ratios of 14:1 at 18 h which had persisted at 24 h (0.5%ID $g^{-1}$ tumour), data consistent with the previous two studies. The biodistribution data generated for [$^{125}$I]TO6D4 also appeared to support the findings of the previous study, with a higher %ID in tumour (1.5%) at 24 h p.i. and lower T/B ratio (4.5:1). Again, the lower kidney:blood ratio for [$^{125}$I]TO6D4 at 24 h suggested a difference from [$^{125}$I]CEA6 in extent of kidney retention. Tumour uptake of [$^{125}$I]TO6D12 (0.4%) was similar to that obtained with [$^{125}$I]CEA6 (Table 2) but, in this study, the T/B ratio appeared to be lower than that measured previously (6.6:1 as compared with 11:1).

### DISCUSSION

We have previously described the isolation by phage display of a CEA specific scFv, CEA6, from a large unimmunized human library (Vaughan et al, 1996). The dissociation constant of CEA6 for CEA is in the nanomolar range (7.7 × $10^{-9}$ M), which is typical of antibodies isolated from very large repertoires (Perelson and Oster, 1979; Griffiths et al, 1994; Vaughan et al, 1996). Our early work showed that the antibody binds CEA-expressing cells by flow cytometry and immunocytochemistry, and localizes to CEA-expressing human tumour xenografts in nude mice. Because CEA6 had fulfilled the specificity requirements as a potential human tumour targeting agent, it was subjected to affinity maturation using a combination of low redundancy mutagenesis in CDR3 of heavy and light chains and light chain shuffling (Osbourn et al, 1996). Selection for clones with longer off-rates ($K_{off}$) than CEA6 was achieved by solution selection in the presence of excess free antigen (Hawkins et al, 1992).

Our previous work reported the antigenic specificity and binding kinetics of the CEA6-derived mutants (Osbourn et al, 1996). Our longer term objective was to determine whether the off-rate component of affinity would influence the amount of antibody associated with tumour at different times after intravenous administration. We carried out the in vivo experiments with CEA6 and all five mutant scFv (HBB11, TO6D10, TO6D4, TO6D12 and TO6D11), spanning a range of $K_{off}$ values from 6.2 × $10^{-3}$ $s^{-1}$ to 9.0 × $10^{-4}$ $s^{-1}$. To eliminate the role of avidity effects in the biodistribution profiles, purified monomeric fractions of scFv were used for the experiments, because we and others have observed a tendency of scFv to form dimers (Griffiths et al, 1992).

One of the advantages of using $^{125}$I as the labelling radioisotope is that the IodoGen method allows more than one atom of iodine to be introduced per scFv, thereby increasing the sensitivity of detection. Labelling of scFv with Technetium-99m at single specific sites remote from the binding domains has been successful for imaging applications (Liberatore et al, 1995; Verhaar et al, 1996) but is less sensitive than iodine. However, one disadvantage of iodination is that it modifies tyrosine residues including those in the antigen binding site. CEA6 and the mutants derived from it have at least 14 tyrosine residues available for attachment of iodine, some of which are in complementarity-determining regions, yet in the affinity chromatography experiments described here, none of the clones showed reduced CEA binding specificity following radioiodination. This was subsequently confirmed in their tumour localization profiles.

**Figure 5** (**A**) Mean %ID $g^{-1}$ of tissue 24 h post i.v. administration of [$^{125}$I]CEA6 (filled bars), [$^{125}$I]TO6D10 (diagonal stripe bars), [$^{125}$I]TO6D4 (open bars), [$^{125}$I]TO6D12 (horizontal stripe bars) and [$^{125}$I]HBB11 (vertical stripe bars) to LS174T xenografted mice. Mean values are derived from $n = 4$ mice and vertical bars indicate standard deviation (s.d.) from the mean. (**B**) Mean tissue:blood ratio 24 h post i.v. administration of [$^{125}$I]CEA6, [$^{125}$I]TO6D10, [$^{125}$I]TO6D4, [$^{125}$I]TO6D12 and [$^{125}$I]HBB11 to LS174T xenografted mice

Rapid fractional clearance of murine scFv from the blood after intravenous administration has often been reported to be the determining factor in the concentration of scFv taken up into tumour (Colcher et al, 1990; Milenic et al, 1991; Savage et al, 1993). For all $^{125}$I-labelled scFv examined in this study, clearance of the bulk of the injected dose was primarily via the kidney at early time points. There was no significant uptake of radioactivity into any of the normal tissues at the time points tested, although the degree of kidney retention over time varied from clone to clone. The lower tumour–kidney ratio at 24 h, measured for scFv TO6D4, suggested a rate of clearance more typical of Fab′ or F(ab′)2 fragments (Colcher et al, 1990; Rowlinson-Busza et al, 1996). However, whole-body clearance rates determined for TO6D4 in parallel with TO6D12 and CEA6 subsequently showed indistinguishable $t_{1/2}$ measurements of approximately 2 h (data not shown).

All scFv were preferentially localized in tumour 6 h after injection, and by 24 h the amount of the injected dose retained at the tumour site varied from one scFv to another. The scFv with the lowest $K_{off}$ TO6D11 did not show the highest T/B ratio at 24 h, and it was in fact CEA6 which gave the most consistent performance in the

 © Cancer Research Campaign 1998

**Table 2** Comparison of biodistribution of CEA6, TO6D12 and TO6D4 scFv

| | 3 h | | 6 h | | 18 h | | 24 h | |
|---|---|---|---|---|---|---|---|---|
| CEA 6 | | | | | | | | |
| Blood | 4.20 ± 1.37 | [1.0] | 2.23 ± 0.43 | [1.0] | 0.09 ± 0.01 | [1.0] | 0.05 ± 0.02 | [1.0] |
| Liver | 1.74 ± 0.50 | [0.4] | 2.62 ± 2.70 | [1.4] | 0.08 ± 0.02 | [0.9] | 0.06 ± 0.01 | [1.3] |
| Kidney | 9.99 ± 3.30 | [2.4] | 2.20 ± 0.31 | [1.0] | 0.26 ± 0.14 | [2.6] | 0.14 ± 0.02 | [3.5] |
| Lung | 3.00 ± 0.91 | [0.7] | 1.66 ± 0.38 | [0.7] | 0.08 ± 0.02 | [0.9] | 0.06 ± 0.01 | [1.5] |
| Spleen | 1.86 ± 0.52 | [0.5] | 1.06 ± 0.17 | [0.5] | 0.10 ± 0.05 | [1.1] | 0.07 ± 0.01 | [1.7] |
| Colon | 2.06 ± 0.73 | [0.5] | 1.06 ± 0.25 | [0.5] | 0.07 ± 0.02 | [0.8] | 0.06 ± 0.02 | [1.3] |
| Muscle | 1.26 ± 0.57 | [0.3] | 0.53 ± 0.10 | [0.2] | 0.05 ± 0.02 | [0.5] | 0.03 ± 0.01 | [0.8] |
| Femur | 1.87 ± 0.65 | [0.4] | 0.97 ± 0.22 | [0.4] | 0.07 ± 0.02 | [0.8] | 0.06 ± 0.01 | [1.4] |
| Tumour | 5.78 ± 1.14 | [1.4] | 2.95 ± 0.91 | [1.4] | 1.36 ± 0.76 | [14.0] | 0.46 ± 0.02 | [14.6] |
| TO6D12 | | | | | | | | |
| Blood | 3.54 ± 1.01 | [1.0] | 2.65 ± 0.62 | [1.0] | 0.07 ± 0.03 | [1.0] | 0.05 ± 0.02 | [1.0] |
| Liver | 1.58 ± 0.49 | [0.4] | 1.19 ± 0.34 | [0.5] | 0.08 ± 0.02 | [1.1] | 0.05 ± 0.01 | [1.1] |
| Kidney | 4.65 ± 1.32 | [1.3] | 2.41 ± 0.40 | [0.9] | 0.20 ± 0.05 | [2.9] | 0.14 ± 0.03 | [2.7] |
| Lung | 2.39 ± 0.61 | [0.7] | 1.84 ± 0.42 | [0.7] | 0.09 ± 0.03 | [1.2] | 0.06 ± 0.01 | [1.2] |
| Spleen | 1.57 ± 0.38 | [0.5] | 1.19 ± 0.29 | [0.5] | 0.07 ± 0.02 | [1.1] | 0.07 ± 0.03 | [1.6] |
| Colon | 1.58 ± 0.45 | [0.5] | 2.40 ± 1.07 | [0.9] | 0.06 ± 0.02 | [0.9] | 0.04 ± 0.01 | [0.9] |
| Muscle | 0.99 ± 0.32 | [0.3] | 0.70 ± 0.12 | [0.3] | 0.05 ± 0.02 | [0.7] | 0.04 ± 0.01 | [0.8] |
| Femur | 1.61 ± 0.34 | [0.5] | 1.13 ± 0.19 | [0.4] | 0.07 ± 0.02 | [1.1] | 0.07 ± 0.01 | [1.5] |
| Tumour | 4.17 ± 1.34 | [1.2] | 3.24 ± 0.71 | [1.2] | 0.40 ± 0.12 | [5.9] | 0.35 ± 0.15 | [6.6] |
| TO6D4 | | | | | | | | |
| Blood | 6.84 ± 1.10 | [1.0] | 3.17 ± 1.07 | [1.0] | 0.37 ± 0.02 | [1.0] | 0.35 ± 0.06 | [1.0] |
| Liver | 2.95 ± 0.53 | [0.4] | 1.41 ± 0.31 | [0.5] | 0.19 ± 0.01 | [0.5] | 0.20 ± 0.03 | [0.6] |
| Kidney | 7.96 ± 1.41 | [1.2] | 2.95 ± 1.00 | [0.9] | 0.53 ± 0.04 | [1.5] | 0.46 ± 0.02 | [1.4] |
| Lung | 4.11 ± 0.67 | [0.6] | 1.88 ± 0.65 | [0.6] | 0.25 ± 0.05 | [0.7] | 0.23 ± 0.05 | [0.7] |
| Spleen | 2.48 ± 0.40 | [0.4] | 1.30 ± 0.41 | [0.4] | 0.16 ± 0.01 | [0.4] | 0.17 ± 0.03 | [0.5] |
| Colon | 2.08 ± 0.28 | [0.3] | 1.04 ± 0.42 | [0.3] | 0.08 ± 0.01 | [0.2] | 0.09 ± 0.03 | [0.3] |
| Muscle | 1.04 ± 0.22 | [0.2] | 0.59 ± 0.36 | [0.2] | 0.05 ± 0.004 | [0.2] | 0.04 ± 0.003 | [0.1] |
| Femur | 1.71 ± 0.34 | [0.3] | 0.76 ± 0.30 | [0.2] | 0.10 ± 0.01 | [0.3] | 0.08 ± 0.02 | [0.2] |
| Tumour | 8.32 ± 1.40 | [1.2] | 4.54 ± 1.43 | [1.5] | 2.69 ± 1.39 | [7.4] | 1.53 ± 0.22 | [4.5] |

Mean %ID/g of tissue at specified time points after i.v. administration of [$^{25}$I]CEA6, [$^{25}$I]TO6D12 and [$^{25}$I]TO6D4 scFv to LS174T tumour xenograft-bearing mice. Mean values are derived from $n$=4 mice ± standard deviation (s.d.) from mean. Mean tissue-blood ratios are given in square parentheses and are determined from the mean %ID/g of the tissues divided by the %ID/g in blood.

three experiments, with T/B ratios as high as 22:1 at 24 h. It is possible that subtle differences in biodistribution do indeed exist between individual antibodies in the panel, and even that the difference correlates with off-rate, as TO6D11 showed improved tumour retention at 48 h relative to CEA6; however, one could argue that the improved T/B ratio for TO6D11 at later timepoints could be due to reduced kidney retention. Furthermore, it was not possible in these experiments to demonstrate a gradient of prolonged tumour residence time corresponding directly to the respective off-rate of the scFv. Of additional relevance to this is the effect of tumour antigen concentration on the degree of antibody localization: in the LS174T tumour xenograft model, the abundant expression of CEA may exceed the concentration at which increasing the affinity could have a measurable effect. This would not be expected in colorectal tumours in man, which show large individual variations in CEA concentration.

Because the differences in off-rate between the clones are all within an order of magnitude and are measured against a background of rapid pharmacokinetic clearance, it may not be possible to establish a tumour targeting ranking for clones in scFv format. Reformatting the scFv as higher molecular weight proteins, or using chemical modification to increase their residence time in vivo, could be ways of analysing the influence of off-rate more closely. The majority of studies in the literature correlating affinity with tumour uptake have been carried out with intact monoclonal antibodies, or fragments derived by proteolytic digestion that have a longer serum half-life than scFv. In a study of the uptake of rodent mAbs into CEA-expressing tumours in humans, Sharkey et al (1993) concluded that a high-affinity mAb would achieve improved tumour uptake and longer residence time at the tumour. However, they also conceded that the bulk of an injected high affinity antibody would be absorbed by CEA shed in the serum and by cells at the tumour periphery, whereas a lower affinity mAb would have the chance to penetrate to CEA positive cells deeper in the tumour and thereby result in a better therapeutic agent.

As well as the different patterns of in vivo behaviour of the panel of scFv, flow cytometry has revealed differences in extent of binding to a human liver cell line: CEA6, HBB11 and, to a very small extent, TO6D12 were partially cross-reactive with Chang cells, while the remaining affinity-matured clones were not. Although structurally related markers of the CEA superfamily are known to be expressed on liver (Hinoda et al, 1988), the binding of CEA6, HBB11 and TO6D12 cannot be explained as cross-reactivity to such molecules, as normal human liver stained negative by immunocytochemistry for all of the scFv in the study. It is not possible to explain the cross-reactive component in these antibodies on the basis of sequence, as there is no light chain sequence consensus between CEA6, HBB11 and TO6D12 that distinguishes them from the other clones (Osbourn et al, 1996). Likewise, the extent of liver cell binding does not appear to increase with increasing affinity for CEA. However, because we had not observed increased liver retention of [$^{125}$I]CEA6, [$^{125}$I]HBB11 or [$^{125}$I]TO6D12 in mice, the results suggest that the element of cross-reactivity is only relevant to a subcomponent of Chang human liver cells, through the binding of an unknown cell-surface antigen.

It has recently been shown that a radiolabelled scFv specific for human CEA is a potent tumour imaging agent, achieving exceptional T/B ratios in patients (Begent et al, 1996). The scFv in question,

© Cancer Research Campaign 1998 

MFE23, was isolated from a phage display library constructed from a CEA-immunized mouse and has an affinity for CEA of $2.5 \times 10^{-9}$ M (Chester et al, 1994). Our work demonstrates that CEA6 compares very favourably with MFE23 in a mouse tumour xenograft model and has an affinity for CEA in the same range. However, one clear distinction between the two antibodies is that while MFE23 was derived from antigenic selection in a mouse, CEA6 has been selected directly from a non-immunized human library and functions in tumour targeting without additional affinity maturation. This study has shown that CEA6 has all of the essential properties for reformatting as a higher molecular weight molecule to prolong its in vivo half-life and thereby improve its performance as a fully human therapeutic agent. These studies are currently in progress.

### ACKNOWLEDGEMENTS

We would like to thank R Boden for technical assistance and Professor R Begent and J McCafferty for critical reading of the manuscript.

### REFERENCES

Begent RHJ, Verhaar MJ, Chester KA, Casey JL, Green AJ, Napier MP, Hope-Stone LD, Cushen N, Keep PA, Johnson CJ, Hawkins RE, Hilson AJW and Robson L (1996) Clinical evidence of efficient tumor targeting based on single-chain Fv antibody selected from a combinatorial library. *Nature Med* **2**: 979–984

Chester KA, Begent RHJ, Robson L, Keep P, Pedley RB, Boden JA, Boxer G, Green A, Winter G, Cochet O and Hawkins RE (1994) Phage libraries for generation of clinically useful antibodies. *Lancet* **343**: 455–456

Colcher D, Bird R, Roselli M, Hardman KD, Johnson S, Pope S, Dodd SW, Pantoliano MW, Milenic DE and Schlom J (1990) *In vivo* tumor targeting of a recombinant single-chain antigen-binding protein. *J Natl Cancer Inst* **82**: 1191–1197

Goldenberg DM, Goldenberg H, Sharkey RM, Lee RE, Horowitz JA, Hall TC and Hansen HJ (1990) *In vivo* antibody imaging for the detection of human tumors. In *Cancer Imaging with Radiolabeled Antibodies*, Goldenberg DM (ed), pp. 273–292, Kluwer Academic Publishers: Dordrecht

Griffiths AD, Malmqvist M, Marks JD, Bye JM, Embleton MJ, McCafferty J, Baier M, Holliger KP, Gorick BD, Hughes-Jones, NC, Hoogenboom HR and Winter G (1993) Human anti-self antibodies with high specificity from phage display libraries. *EMBO J* **12**: 725–734

Griffiths AD, Williams SC, Hartley O, Tomlinson IM, Waterhouse P, Crosby WL, Kontermann RE, Jones PT, Low NM, Allison TJ, Prospero TD, Hoogenboom HR, Nissim A, Cox JPL, Harrison JL, Zaccolo M, Gherardi E and Winter G (1994) Isolation of high affinity human antibodies directly from large synthetic repertoires. *EMBO J* **13**: 3245–3260

Harwood PJ, Boder J, Pedley RB, Rauling G, Rogers GT and Bagshawe KD (1985) Comparative tumor localization of antibody fragments and intact IgG in nude mice bearing CEA producing human colon carcinoma xenografts. *Eur J Cancer Clin Oncol* **21**: 1515–1522

Hawkins RE, Russell SJ and Winter G (1992) Selection of phage antibodies by binding affinity: mimicking affinity maturation. *J Mol Biol* **226**: 889–896

Hinoda Y, Neumaier M, Hefta SA, Drzeniek Z, Wagener C, Shively L, Hefta LJF, Shively JE and Paxton RJ (1988) Molecular cloning of a cDNA coding biliary glycoprotein 1: primary structure of a glycoprotein immunologically crossreactive with carcinoembryonic antigen. *Proc Natl Acad Sci USA* **85**: 6959–6963

Hu S-z, Shively L, Raubitschek A, Sherman M, Williams LE, Wong JYC, Shively JE and Wu AM (1996) Minibody: a novel engineered anti-carcinoembryonic antigen antibody fragment (single-chain Fv-CH3) which exhibits rapid, high-level targeting of xenografts. *Cancer Res* **56**: 3055–3061

Huston JS, McCartney J, Tai M-S, Mottola-Hartshorn C, Jin D, Warren F, Keck P and Oppermann H (1993) Medical applications of single-chain antibodies. *Intern Rev Immunol* **10**: 195–217

Johnson KS and Chiswell DJ (1993) Human antibody engineering. *Curr Opinion Structural Biol* **3**: 564–571

King DJ, Turner A, Farnsworth APH, Adair JR, Owens RJ, Pedley RB, Baldock D, Proudfoot KA, Lawson ADG, Beeley NRA, Millar K, Millican TA, Boyce BA, Antoniw P, Mountain A, Begent RHJ, Shochat D and Yarranton GT (1994) Improved tumor targeting with chemically cross-linked recombinant antibody fragments. *Cancer Res* **54**: 6176–6185

Langmuir VK, Mendonca HL and Woo DV (1992) Comparisons between two monoclonal antibodies that bind to the same antigen but have differing affinities: uptake kinetics and $^{125}$I-antibody therapy efficacy in multicell spheroids. *Cancer Res* **52**: 4728–4734

Liberatore M, Neri D, Neri G, Pini A, Iurilli AP, Ponzo F, Spampinato G, Padula F, Pala A and Colella AC (1995) Efficient one-step labelling of recombinant antibodies with technetium-99m. *Eur J Nucl Med* **22**: 1326–1329

Milenic DE, Yokota T, Filpula DR, Finkelman MAJ, Dodd SW, Wood JF, Whitlow M, Snoy P and Schlom J (1991) Construction, binding properties, metabolism and tumor targeting of a single chain Fv derived from the pancarcinoma monoclonal antibody CC49. *Cancer Res* **51**: 6363–6371

Munro S and Pelham HRB (1986) An hsp70-like protein in the ER: identity with the 78 kDa glucose-regulated protein and immunoglobulin heavy chain binding protein. *Cell* **46**: 291–300

Osbourn JK, Field A, Wilton J, Derbyshire E, Earnshaw JC, Jones PT, Allen D and McCafferty J (1996) Generation of a panel of related human scFv antibodies with high affinities for human CEA. *Immunotechnology* **2**: 181–196

Parsons HL, Earnshaw JC, Wilton J, Johnson KS, Schueler PA, Mahoney W and McCafferty J (1996) Directing phage selection towards specific epitopes. *Prot Eng* **9**: 1043–1049

Pedley RB, Boden JA, Boden R, Dale R and Begent RH (1993) Comparative radioimmunotherapy using intact or $F(ab')_2$ fragments of $^{131}$I anti-CEA antibody in a colonic xenograft model. *Br J Cancer* **68**: 69–73

Perelson AS and Oster GF (1979) Theoretical studies of clonal selection: minimal antibody repertoire size and reliability of self non-self discrimination. *J Theor Biol* **81**: 645–670

Pimm MV and Gribben SJ (1993) Influence of syngeneic (anti-idiotypic) antibody responses on biodistribution and tumour localisation of murine monoclonal antibodies and fragments. *Anticancer Res* **13**: 241–248

Rowlinson-Busza G, Deonarain MP and Epenetos AA (1996) Comparison of intact monoclonal antibody, its F(ab')2 and Fab fragments and recombinant single chain Fv in a human tumor xenograft model. *Tumor Targeting* **2**: 37–48

Savage P, Rowlinson-Busza G, Verhoeyen M, Spooner RA, So A, Windust J, Davis PJ and Epenetos AA (1993) Construction, characterisation and kinetics of a single chain antibody recognising the tumour associated antigen placental alkaline phosphatase. *Br J Cancer* **68**: 738–742

Schier R, McCall A, Adams GP, Marshall KW, Merritt H, Yim M, Crawford RS, Weiner LM, Marks C and Marks JD (1996) Isolation of picomolar affinity anti-c-erbB-2 single-chain Fv by molecular evolution of the complementary determining regions in the center of the antibody binding site. *J Mol Biol* **263**: 551–567

Schlom J, Eggensperger D, Colcher D, Molinolo A, Houchens D, Miller LS, Hinkle G and Siler K (1992) Therapeutic advantage of high-affinity anticarcinoma radioimmunoconjugates. *Cancer Res* **52**: 1067–1072

Sharkey RM, Goldenberg DM, Murthy S, Pinsky H, Vagg R, Pawlyk D, Siegal JA, Wong GY, Gascon P, Izon DO, Vezza M, Burger K, Swayne LC, Pinsky CM and Hansen HJ (1993) Clinical evaluation of tumor targeting with a high affinity, anticarcinoembryonic antigen-specific murine monoclonal antibody, MN14. *Cancer* **71**: 2082–2096

Thompson J, Pope T, Tung J-S, Chan C, Hollis G, Mark G and Johnson KS (1996) Affinity maturation of a high affinity human monoclonal antibody against the third hypervariable loop of human immunodeficiency virus: use of phage display to improve affinity and broaden strain reactivity. *J Mol Biol* **256**: 77–88

Vaughan T, Williams AJ, Pritchard K, Osbourn JK, Pope AR, Earnshaw JC, McCafferty J, Wilton J and Johnson KS (1996) Human antibodies with sub-nanomolar affinities isolated from a large non-immunised phage display library. *Nature Biotechnol* **14**: 309–314

Verhaar MJ, Chester KA, Keep PA, Robson L, Pedley RB, Boden JA, Hawkins RE and Begent RHJ (1995) A single chain Fv derived from a filamentous phage library has distinct tumor targeting advantages over one derived from a hybridoma. *Int J Cancer* **61**: 497–501

Verhaar MJ, Keep PA, Hawkins RE, Robson L, Casey JL, Pedley B, Boden JA, Begent RHJ and Chester KA (1996) $^{99m}$Tc radiolabelling using a phage-derived single chain Fv with C-terminal cysteine for colorectal tumour imaging. *J Nucl Med* **37**: 68–72

Yokota T, Milenic DE, Whitlow M and Schlom J (1992) Rapid tumour penetration of a single-chain Fv and comparison with other immunoglobulin forms. *Cancer Res* **52**: 3402–3408

© Cancer Research Campaign 1998